# Persistent inequalities in global air quality monitoring should not delay pollution mitigation

**DOI:** 10.1073/pnas.2423259122

**Published:** 2025-04-28

**Authors:** E. Keith Smith, Camille Fournier de Lauriere, Ella Henninger

**Affiliations:** ^a^Department of Humanities, ETH Zurich, Social and Political Sciences, International Political Economy and Environmental Politics, Zurich 8092, Switzerland

**Keywords:** air pollution, monitoring capacity, barrier to mitigation, “no regrets” policies

## Abstract

Air pollution is a global health crisis that disproportionately affects lower- and middle-income countries. We examine global air quality monitoring data, highlighting persistent inequalities in high-exposure regions, and assess the potential of emerging technologies to improve data utility for mitigation. Improving air quality, however, does not need to wait for more data, nor will monitoring alone drive action. Immediate mitigation measures with broad cobenefits are needed now to reduce pollution, even in data-scarce settings.

Air pollution is a leading global health threat, disproportionately affecting densely populated urban areas in lower- and middle-income countries (1). Sociopolitical efforts to improve localized air quality are difficult to achieve. A lack of air quality data is frequently cited as a barrier to mitigation efforts, as summarized summarized by a recent New York Times opinion, “data—or, more precisely, a lack of data is the most immediate problem... when air quality data is available, pollution declines” (2). Emerging technologies have greatly increased globalized monitoring capacity, but substantial data gaps persist (3). Even to this day, we might not know which city is the most polluted worldwide (4).

Here, we evaluate the state of global air quality monitoring, identifying gaps, and assessing emerging technologies. Further, we critically examine whether generating more data, on its own, can reduce air pollution. We highlight the opportunity costs of prioritizing monitoring over mitigation, and discuss how increasing monitoring without concurrent mitigation risks unintended policymaking consequences. In locales where monitoring remains sparse, rather than waiting for more data before taking action, governments should implement “no regrets” policies now to reduce pollution while delivering economic, infrastructure, and public health cobenefits.

## The Promise of Emerging Data Sources.

Historically, air quality–monitoring data have been rather scarce in lower- and middle-income countries, largely due to high costs and maintenance demands of traditional “regulatory-grade” systems. Recent years have seen significant growth in monitoring capacity, driven by emerging technologies like satellite-based remote sensing, with continually improving spatial and temporal resolutions (5). Nevertheless, current uncertainties in these modeled pollution estimates, especially in countries that lack substantial on-the-ground regulatory-grade measurements, limit their utility to support mitigating activities. “Nonregulatory” devices, such as “low cost” sensors, have further expanded on-the-ground monitoring at a fraction of the cost and with significantly less infrastructure requirements (power and internet) compared to regulatory-grade monitors. Though promising, nonregulatory grade monitors require technical improvements [enhanced chemical detection, accuracy (6)] and regulatory integration [adoption into enforcement frameworks (7)] to match regulatory-grade standards.

## Inequalities Persist in Air Quality Monitoring.

**Fig. 1.**
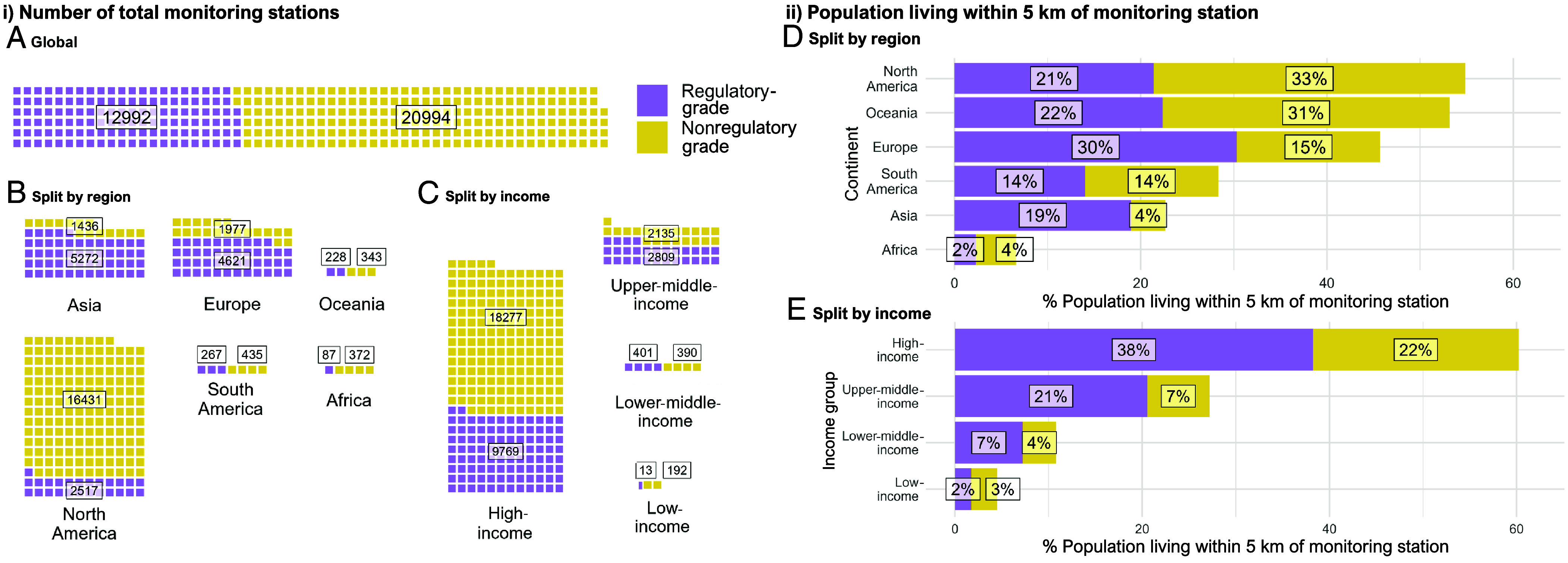
Worldwide distribution of on-the-ground monitoring stations reporting in April 2024. Each full unit (square) represents 100 monitoring stations, with regulatory-grade monitors in purple and nonregulatory grade monitors in yellow. Panel (*A*) describes all global monitoring, Panel (*B*) presents monitoring by geographical region, and Panel (*C*) displays monitoring by country-level World Bank income groups. Panel (*D*) shows the proportion of the population living less than 5 km away from a monitoring station by region and Panel (*E*) by country-level World Bank income groups.

Even in the era of increased globalized monitoring, large information deserts persist. We developed a harmonized database of on-the-ground air quality monitoring stations, categorized by the monitoring type (regulatory/nonregulatory grade), geographic location, and level of economic development (Fig. 1). Approximately 95% of the 33,984 air quality monitoring stations are in Asia, Europe, or North America, while less than 4% are located in Africa or South America (Fig. 1*B*). Similarly, only 3% of global monitoring stations are located in either lower- or lower-middle-income countries (Fig. 1*C*).

Globally, 62% of monitoring stations are nonregulatory grade, with North America contributing significantly. North America contains 78% of global nonregulatory grade monitors, which constitutes 87% of all stations within this region. Contrastingly, the proportion of nonregulatory grade monitors is much lower in Asia (21%) and in Europe (30%). Locales with fewer total monitoring stations are more likely to rely upon nonregulatory grade devices. Currently, 81% of all stations in Africa are nonregulatory grade, while 94% of stations in lower income countries are nonregulatory grade.

The proportion of people living within 5 km of any monitoring station, regulatory or nonregulatory (Fig. 1*D*) is highest in North America (54%), Oceania (53%), and Europe (45%), compared to 23% in Asia and 6% in Africa. People in high-income countries (Fig. 1*E*) are approximately 12 times more likely to live within 5 km of a monitoring station than those in low-income countries (60% vs. 5%), and 6 times more likely than those in lower-middle-income countries (60% vs. 11%).

## Enhancing Capacity, Validity, and Utility of Emerging Monitoring Technologies.

Currently, data from nonregulatory grade monitors is rather heterogeneous and broadly considered inferior to regulatory-grade systems. A recent United States Government Accountability Office report (7) suggests several policy options to support emerging nonregulatory grade monitoring technologies (supporting innovation, development of standards and compliance testing, improved data management and sharing best practices, and collaborative dissemination of expert knowledge to stakeholders). Increased data quality and comparability can enable nonregulatory grade monitors to support legal compliance and enforcement—key factors driving effective localized implementation of air quality measures. In lower- and middle-income countries, supporting regionally developed nonregulatory grade monitors calibrated to local climates can improve the quantity and validity of monitoring. Currently, several organizations are pioneering efforts across Africa, such as the AirQo network and the Afri-SET collaboration.

New air quality data should be open, transparent, and integrate health, economic, and behavioral data. Such efforts enable assessment of both unequal pollution exposure, as well as the impacts on hindering development and healthcare costs. Many mitigation measures, like rural electrification, mass transit, waste management, and clean energy, align with sustainable development goals. Integrating air quality, health, and economic data can guide policies that deliver broad, multidimensional benefits.

Prioritizing the collection of better data on specific pollutants’ characteristics is more beneficial than simply collecting more data on common metrics. PM_2.5_ and PM_10_ measure particle size, but their chemical composition, pollutant mix, and toxicity vary significantly by location (8). Understanding pollutant composition enhances source attribution, health assessments, mitigation strategies, and allocation, especially in resource-limited settings.

## Improving Air Quality Does Not Have to Wait for More Data.

Enhanced air quality monitoring can support mitigation by raising public awareness, amplifying citizen demands, and increasing prioritization (3, 9). Monitoring supports identifying pollution sources, targeted mitigation measures, and effectiveness evaluations, making it a key component of phased strategies like Air Quality Management Plans. However, while precise data are often unavailable, prima facie evidence of poor air quality is undeniable, and necessitating monitoring before acting can delay mitigation and cause unintended risks.

Prioritizing monitoring over mitigation efforts incurs opportunity costs. Many existing solutions, such as improved fuel quality and vehicle emission standards, are well established and do not require preexisting monitoring (10). In lower- and middle-income countries, mandating monitoring before mitigation measures can create economic and logistical burdens, prolonging pollution exposure. Regardless of existing monitoring capacity, governments can implement “no regrets” policies (11), such as improved waste management, electrification, road infrastructure, public transit, and renewable energy investments, which provide needed economic and health cobenefits in addition to pollution control. Further, framing mitigation as development-focused, rather than environmental, can enhance political feasibility.

While air quality data can raise awareness, knowledge alone rarely drives policy or behavioral change. Governments already face “burden-capacity gaps” (12), where existing policies often remain unenforced. Greater administrative capacity is required, not just more data, to implement regulations already “on the books” (open waste burning bans, vehicle emissions testing, industrial pollution controls). While at the individual level, a multitude of factors hinder behavioral changes, such as the “knowledge-behavior gap,” low trust (13) and limited perceived “response efficacy” (14).

Pollution mitigation measures should be developed alongside monitoring. But increasing monitoring activity uncoupled from substantive, concurrent mitigation measures carries potential risks and unintended consequences. In such cases, governments, responding to mounting public pressure, may opt for shorter-term, attention-grabbing ‘gimmicks’ at the expense of longer-term, effective solutions. For example, “anti-smog towers” (15) or cloud seeding are often criticized as symbolic politics, and are neither effective nor practical mitigation solutions. For individuals, increasing awareness without visible action can lead to a sense of fatalism, or the belief that nothing can improve air pollution, making them less likely to take action or support policies.

Ultimately, the lack of data should not necessitate a lack of action. Waiting for comprehensive, or more “perfect” data, risks worsening health outcomes for those currently exposed to air pollution. Improving air quality requires concrete mitigating actions, regardless of data availability.

## Materials and Methods

The harmonized country-level dataset includes 33,986 regulatory and nonregulatory grade monitoring stations that reported data in April 2024 from either AirNow, AirQo, OpenAQ, PurpleAir, or WAQI APIs [Zenodo (DOI: 10.5281/zenodo.14056196) (16)]. Stations are aggregated by continent and country income levels (World Bank 2024 classification). Population proximity to monitoring stations was estimated using 2020 gridded data. Expanded methodological details are presented in *SI Appendix*.

## Supplementary Material

Appendix 01 (PDF)

## Data Availability

Replication data and code have been deposited in Zenodo (3).

## References

[1] J. Rentschler, N. Leonova, Global air pollution exposure and poverty. *Nat. Commun.* **14**, 4432 (2023). 10.1038/s41467-023-39797-4 PMC10363163 37481598

[2] C. Hasenkopf, Opinion One of the World’s Biggest Health Risks Is a Philanthropic Blind Spot (The New York Times, 2024).

[3] M Schoch, C Fournier de Lauriere, T Bernauer, Monitoring Urban Air Pollution in the Global South: Large Gaps Associated with Economic Conditions and Political Institutions. bioRxiv [Preprint] (2025). 10.1101/2025.03.06.641972 (Accessed 5 April 2025). PMC12798989 41528976

[4] R. V. Martin , No one knows which city has the highest concentration of fine particulate matter. Atmos. Environ. X **3**, 100040 (2019).

[5] A. van Donkelaar *et al*., Monthly global estimates of fine particulate matter and their uncertainty. *Environ. Sci. Technol.* **55**, 15287–15300 (2021). 10.1021/acs.est.1c05309 34724610

[6] M. R. Giordano , From low-cost sensors to high-quality data: A summary of challenges and best practices for effectively calibrating low-cost particulate matter mass sensors. J. Aerosol Sci. **158**, 105833 (2021).

[7] United States Government Accountability Office, “Air quality sensors: Policy options to help address implementation challenges” (Tech. Rep. GAO-24-106393, Washington, DC, 2024).

[8] X. Li, L. Jin, H. Kan, Air pollution: A global problem needs local fixes. *Nature* **570**, 437–439 (2019). 10.1038/d41586-019-01960-7 31239571

[9] A. Jha, A. L. Nauze, US Embassy air-quality tweets led to global health benefits. *Proc. Natl. Acad. Sci. U.S.A.* **119**, e2201092119 (2022). 10.1073/pnas.2201092119 PMC9636956 36279451

[10] C. E. Ivey, J. Pruitt, J. Garcia, Comment on “State-of-the-science data and methods need to guide place-based efforts to reduce air pollution inequity”. *Environ. Health Perspect.* **132**, 038001 (2024). 10.1289/EHP14540 PMC10956663 38512317

[11] Intergovernmental Panel on Climate Change (IPCC), *Climate Change 2001: Mitigation. Contribution of Working Group III to the Third Assessment Report of the Intergovernmental Panel on Climate Change* (Cambridge University Press, Cambridge, UK, 2001).

[12] X. Fernández-I-Marín, C. Knill, C. Steinbacher, Y. Steinebach, Bureaucratic quality and the gap between implementation burden and administrative capacities. Am. Polit. Sci. Rev. **118**, 1240–1260 (2024).

[13] B. L. Hoffmann, C. Scartascini, F. G. Cafferata, How can we improve air pollution? Try increasing trust first. *Environ. Dev. Econ.* **27**, 393–413 (2022).

[14] K. Mehiriz, P. Gosselin, The effect of perceived threats and response efficacy on adaptation to smog: An instrumental variables design. *Risk Anal.* **42**, 1042–1055 (2022). 10.1111/risa.13814 34424564

[15] S. Guttikunda, P. Jawahar, Can we vacuum our air pollution problem using smog towers? *Atmosphere* **11**, 922 (2020).

[16] C. Fournier de Lauriere, Global air quality monitoring stations. Zenodo. 10.5281/zenodo.15038373. Accessed 5 April 2025.

