# Supplementary material for: Persistent inequalities in global air quality monitoring should not delay pollution mitigation
Source: Proc Natl Acad Sci U S A. 2025 Apr 28;122(18):e2423259122. doi: 10.1073/pnas.2423259122 (PMC12067272; doi:10.1073/pnas.2423259122)
Supplement: Supplementary file 1 — Appendix 01 (PDF) [file pnas.2423259122.sapp.pdf]

Supporting Information for

Persistent inequalities in global air quality monitoring should not delay pollution mitigation

E. Keith Smith, Camille Fournier de Lauriere and Ella Henninger

E. Keith Smith.
E-mail: keith.smith@gess.ethz.ch

This PDF file includes:

Supporting text
SI References

Supporting Information Text

Methods.

Location of Air Quality Monitoring dataset. We created a harmonised dataset of global air quality monitoring stations that reported pollution levels at least one time during the month of April 2024. This included monitoring stations that reported at least one form of a pollutant (e.g., $PM_{2.5}$, PM_{10}, 0_3, CO, NO_x, SO_x, Black Carbon, CH_4 and Ultra Fine Particle), or an Air Quality Index. We collected locations of air quality monitoring stations reported on either OpenAQ[a], WAQI[b], PurpleAir[c], AirNow[d], or AirQo[e] APIs. These 5 repositories contain a widespread set of different forms of monitoring stations. OpenQ and WAQI repositories include both regulatory-grade (largely from monitors labelled as 'government' sources) and nonregulatory grade monitor stations (from different sensor providers). AirNow includes US governmental air quality monitoring (including national and US diplomatic locales), as well as many other national EPA networks (e.g. Canada, Mexico, Japan), and are primarily regulatory-grade monitors. The PurpleAir register contains data from their own nonregulatory grade monitor networks. The AirQo sensor network is primarily on the African continent, with a majority of sensors located in Uganda. This source of sensors is especially valuable, in view of the limited monitoring capacity in Africa.

We also reviewed other potential data sources, but have excluded these from this database due to different rationales. Sensor locations from IQAir were not included in this analysis due to the predominance of indoor sensors in their network, and restrictions to data access (behind a paywall). Additionally, sensor locations from Senstate, Clarity, AirGradient, and other OpenAQ partners were not individually collected, as these sensors are already accessible via the OpenAQ API.

To prevent double counting, locations listed on multiple platforms, we removed points situated less than 100–300 meters apart (accounting for potential inaccuracies in coordinates across different databases). Additionally, we manually removed some monitoring stations from AirNow in Japan because of some systematic error in the placement of locations of monitors that were also listed in OpenAQ and WAQI, but due to these location errors, were not automatically identified as duplicates. Data was collected from the various APIs using Python or R, and the dataset was harmonised and cleaned using R and the terra package (1).

Analytical Strategy. Panel a) of Figure 1 shows the total number of global regulatory and nonregulatory grade monitors. From the location of monitoring stations around the globe, we calculated the number of regulatory-grade monitors and nonregulatory grade monitors in each country. To do that, we used country borders from the Natural Earth dataset and calculated how many locations were within each country border. Then, we linked our country-level dataset to (i) a continental region classification, (ii) an income group classification, and (iii) a total population count dataset. We used the country:continent classification from Natural Earth, the country:income group classification from World Bank 2023 (2), and the country-level population count dataset from the U.S. Census for 2024. Because data for Venezuela was not available in the World Bank 2023 classification, we used the last available classification (2021), 'upper-middle-income', for that country. To produce panel b) and c) of Figure 1, we used these overarching classifications to split the total number of monitoring stations into 6 continents, and 4 income groups, respectively. This simple visualisation allows us to see how the global pool of AQM is unevenly distributed between different continents and income group. Finally, we used gridded population data from (3) to compute the percentage of the population living less than 5 km from a monitoring station, which is reported in panels d) and e). We used a 5 km buffer around our point locations. If two monitoring stations of the same type (regulatory or nonregulatory grade monitors) were less than 5 km away from each other, we counted the population covered by monitoring stations of this type only once for that overlapping area. We calculated this for every country and reported results for different regions and income groups.

Data availability and replication code. While data is reported in aggregated forms (global, continental grouping, income groupings), the replication data is included on the country-level of analysis. This is to allow for different sets of country grouping to be made. The country-level data can be accessed in Zenodo: 10.5281/zenodo.15038373 (4).

Regulatory and Nonregulatory grade Monitors Terminology. regulatory-grade monitors are high-precision instruments designed to provide reliable and accurate measurements of air pollutants. These regulatory-grade monitors are often installed by national and local governments, and are frequently used to meet regulatory standards. For example, the U.S. EPA (5) has developed standards for Federal Reference Methods (FRMs) or Federal Equivalent Methods (FEMs) to ensure comparability and accuracy of measurements from such monitoring types. regulatory-grade monitoring has high initial costs and ongoing maintenance needs, requiring substantial financial commitment, suitable infrastructure and localised expertise. The data generated from regulatory-grade monitors are largely considered the 'gold standard' for air quality monitoring.

In contrast, nonregulatory grade monitors, often referred to as 'low-cost' sensors or monitors, offer greater flexibility as they require less maintenance and can be deployed in remote environments. However, their limited accuracy prevents their use in regulatory contexts; instead, they are primarily used for personal or community exposure assessments and to be deployed as a network to cover larger areas. We use the term monitoring station for both regulatory and nonregulatory grade monitors.

Both regulatory and nonregulatory grade monitors can measure a wide range of pollutants, such as particulate matter (e.g., PM1/2.5/10), ozone, gases (e.g, NO_2, CO, SO_2, NH_3, H_2S), black carbon, and volatile organic compounds.

[a] https://openaq.org
[b] https://waqi.info
[c] https://www2.purpleair.com
[d] https://www.airnow.gov
[e] https://www.airqo.net

References

1. RJ Hijmans, terra: Spatial Data Analysis (2020) Institution: Comprehensive R Archive Network Pages: 1.8-5.
2. World Bank, World Bank Country and Lending Groups (2024).
3. M Pesaresi, et al., Advances on the Global Human Settlement Layer by joint assessment of Earth Observation and population survey data. *Int. J. Digit. Earth* **17**, 2390454 (2024) Publisher: Taylor & Francis _eprint: https://doi.org/10.1080/17538947.2024.2390454.
4. C Fournier de Lauriere. Global air quality monitoring stations, April 2024 [Data set]. *Zenodo.* https://doi.org/10.5281/zenodo.15038373 (2025). Accessed 05/04/2025.
5. United States Environmental Protection Agency, List of Designated Reference and Equivalent Methods, (Center for Environmental Measurements & Modeling, Washington DC), Technical Report MD-D205-03 (2024).
